# Erucin, the Major Isothiocyanate in Arugula (*Eruca sativa*), Inhibits Proliferation of MCF7 Tumor Cells by Suppressing Microtubule Dynamics

**DOI:** 10.1371/journal.pone.0100599

**Published:** 2014-06-20

**Authors:** Olga Azarenko, Mary Ann Jordan, Leslie Wilson

**Affiliations:** Department of Molecular, Cellular, and Developmental Biology, and the Neuroscience Research Institute, University of California Santa Barbara, Santa Barbara, California, United States of America; CSIC/Universidad Autonoma Madrid, Spain

## Abstract

Consumption of cruciferous vegetables is associated with reduced risk of various types of cancer. Isothiocyanates including sulforaphane and erucin are believed to be responsible for this activity. Erucin [1-isothiocyanato-4-(methylthio)butane], which is metabolically and structurally related to sulforaphane, is present in large quantities in arugula (*Eruca sativa*, Mill.), kohlrabi and Chinese cabbage. However, its cancer preventive mechanisms remain poorly understood. We found that erucin inhibits proliferation of MCF7 breast cancer cells (IC_50_ = 28 µM) in parallel with cell cycle arrest at mitosis (IC_50_ = 13 µM) and apoptosis, by a mechanism consistent with impairment of microtubule dynamics. Concentrations of 5–15 µM erucin suppressed the dynamic instability of microtubules during interphase in the cells. Most dynamic instability parameters were inhibited, including the rates and extents of growing and shortening, the switching frequencies between growing and shortening, and the overall dynamicity. Much higher erucin concentrations were required to reduce the microtubule polymer mass. In addition, erucin suppressed dynamic instability of microtubules reassembled from purified tubulin in similar fashion. The effects of erucin on microtubule dynamics, like those of sulforaphane, are similar qualitatively to those of much more powerful clinically-used microtubule-targeting anticancer drugs, including taxanes and the vinca alkaloids. The results suggest that suppression of microtubule dynamics by erucin and the resulting impairment of critically important microtubule-dependent cell functions such as mitosis, cell migration and microtubule-based transport may be important in its cancer preventive activities.

## Introduction

Epidemiological data have demonstrated that dietary vegetables such as cabbage, cauliflower, kale, arugula, wild rocket and broccoli contain cancer preventive and anti-cancer activities. Isothiocyanates such as sulforaphane and erucin may be responsible for these actions [1], [2]. Several mechanisms are thought to play a role in the cancer preventive activities of isothiocyanates, including inhibition of Phase I carcinogen-activating enzymes [3], [4], induction of Phase II carcinogen detoxification enzymes [5], [6], inhibition of cancer cell proliferation by cell cycle arrest at G_2_/M, and removal of premalignant and malignant cells through the induction of apoptosis [7]–[10].

**Figure 1:**
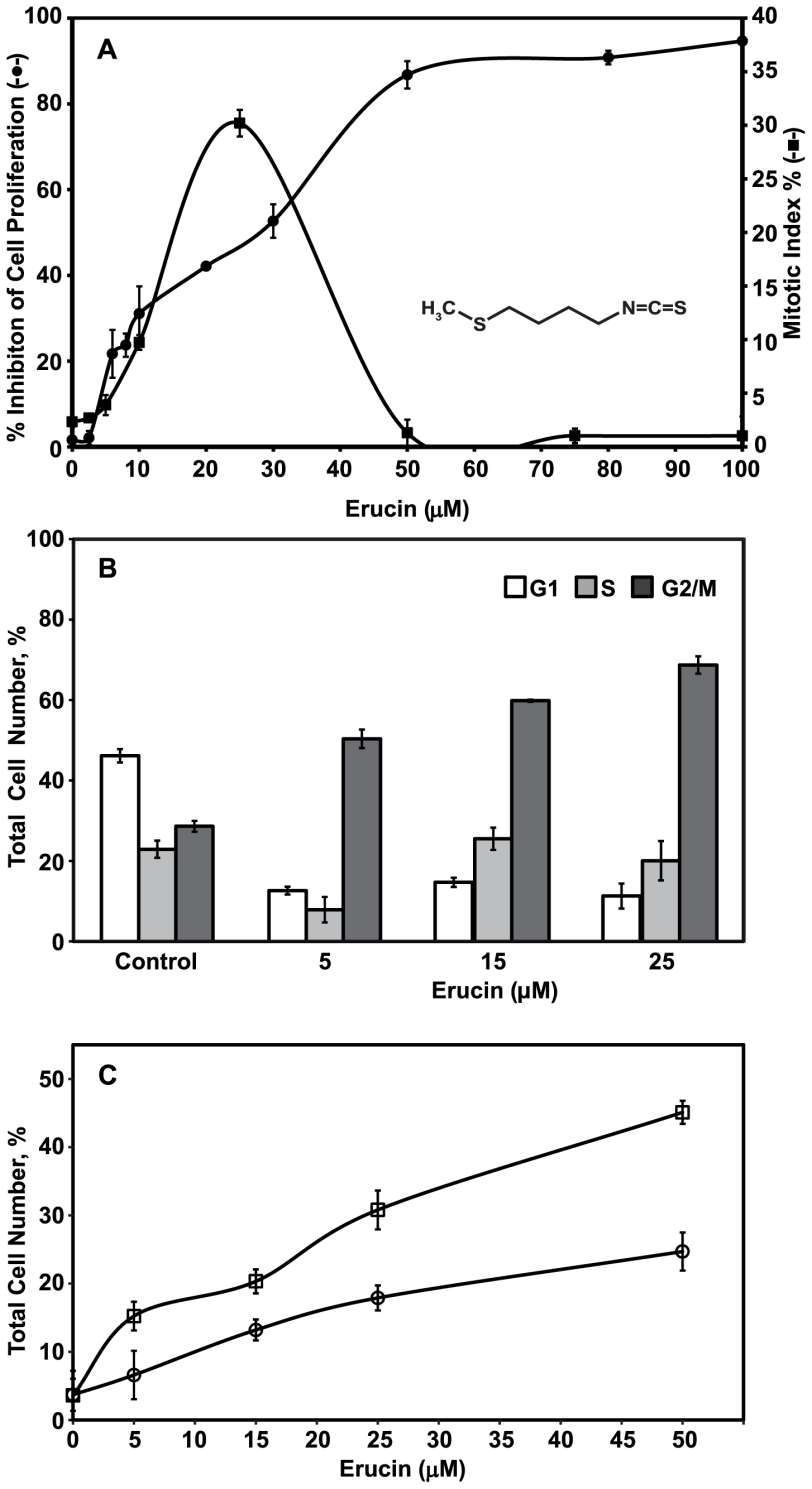
Effects of erucin on MCF7 cell proliferation, mitotic index, cell cycle progression and apoptosis. (**A**) Erucin (inset) inhibits proliferation along with mitotic arrest. Cells were incubated with a range of erucin concentrations for 72 hours and SRB cell proliferation assays were performed to assess cell proliferation (IC_50_ = 28 µM, -•-). To determine the mitotic index (IC_50_ = 13 µM, -▪-), cells were incubated with erucin for 24 hours, fixed, and stained with DAPI to visualize DNA (Materials and Methods). Data are the mean of three to four independent experiments; bars, ± SEM. (**B**) Erucin arrests cells at G2/M. Non-synchronized cells were treated with a range of erucin concentrations for 24 hours and analyzed by flow cytometry (Materials and Methods). White bars represent G1 phase, gray bars, S phase, and black bars, G2/M phase of the cell cycle. (**C**) Erucin induces time-, and concentration-dependent apoptosis. Cells were incubated with a range of erucin concentrations for 24 hours (-○-) and 48 hours (-□-) and the total number of apoptotic cells (early and late apoptotic) for each condition was determined by flow cytometry (Materials and Methods). Results are the mean ± SEM of at least three independent experiments performed in duplicate.

Sulforaphane [1-isothiocyanato-4-(methylsulfinyl)butane] is the most extensively studied isothiocyanate found in cruciferous vegetables. It is especially prominent in broccoli and broccoli sprouts [11]. However, much less is known about the anticancer activity of erucin [1-isothiocyanato-4-(methylthio)butane], a structurally-related sulfide analog of sulforaphane. (Fig. 1, inset). Cruciferous salad crops such as arugula and wild rocket ((*Eruca sativa* (Mill.) and *Diplotaxis tenuifolia* species), several varieties of Chinese cabbage, and kohlrabi, all commonly ingested, accumulate large quantities of glucoerucin [12]–[14], and erucin is produced from this precursor by the endogenous enzyme myrosinase when the vegetables are chopped or chewed. Erucin is also an *in vivo* metabolite of sulforaphane formed through reduction of sulforaphane’s thiomethyl group when various cabbages, especially broccoli and broccoli sprouts, are consumed [15]. Also, the inter-conversion of sulforaphane and erucin is a favored metabolic reaction in animal models and in human subjects who consume either fresh broccoli sprouts or a broccoli supplement [16]–[19]. Dietary isothiocyanates are well absorbed and attain good bioavailability [20]–[22].

Like sulforaphane, erucin induces Phase II detoxification enzymes [23], [24] and inhibits Phase I enzymes [15]. It also has antioxidant activity [25] and increases expression of multidrug resistance transporters in human carcinoma cell lines [26]. In addition, erucin arrests cell cycle progression and induces apoptosis in human lung carcinoma, hepatoma and leukemia cell lines [27]–[30]. These actions may all play a role in erucin’s anticancer actions. The structural similarity between erucin and sulforaphane and the prominence of erucin in several widely-consumed cruciferous vegetables led us to explore the anti-proliferative effects of erucin and its effects on the polymerization and dynamics of microtubules in breast cancer cells and on the dynamics of microtubules reassembled from purified tubulin.

Microtubules are dynamic tube shaped protein polymers (25 nm in diameter) that play important roles in determining cell shape, polarity, cellular migration, signaling, and mitosis [31]–[33]. Microtubules can undergo two unusual non-equilibrium dynamic behaviors, dynamic instability, the switching between growth and shortening at microtubule plus ends [34], [35], and treadmilling, the net plus end assembly and minus end disassembly (reviewed in [31]). Microtubule dynamics are rapid during mitosis and are critical for the accurate and time-sensitive attachment of chromosomes to the mitotic spindle, movement of the chromosomes to form the metaphase plate, and production of proper tension at the kinetochores, all of which are essential for the passage through the metaphase/anaphase spindle checkpoint [36], [37]. Microtubule dynamics are also important in cell polarity, cell migration and metastasis [33].

Many commonly used anticancer drugs act by modulating microtubule dynamics including the taxanes (paclitaxel, docetaxel), the vinca alkaloids (*e.g*., vinblastine, vinorelbine, vincristine), maytansinoids, and eribulin [38], [39]. At a mechanistic level, microtubule-targeting agents act in two ways in cells and *in vitro*. At relatively high concentrations, they either reduce or augment the mass of assembled microtubules. At their lowest effective concentrations, they modulate the growing and shortening dynamics of microtubules without affecting the microtubule polymer mass [38]. Most microtubule-targeting compounds used for treatment of cancer can exert both activities, but as a rule they modulate microtubule dynamics at concentrations significantly lower than those required to increase or decrease the microtubule polymer mass. For example, taxol inhibits proliferation and mitosis in cultured tumor cells in the low nanomolar concentration range by suppressing microtubule dynamics, while the taxol concentration required to significantly increase the microtubule polymer mass is approximately 10 fold higher [40]. Similarly, the vinca alkaloids, which inhibit microtubule polymerization at high concentrations, inhibit cell proliferation at their lowest effective concentrations by altering the dynamics of microtubules without depolymerizing the microtubules [41]. Thus, the most potent actions of these drugs that lead to cell cycle arrest at mitosis and apoptotic cell death in cultured cells are on the dynamics of microtubules, not the quantity of assembled polymer [31], [38], [42].

Recently, we reported that sulforaphane inhibits proliferation and mitosis in MCF7 cells in the micromolar concentration range by suppressing microtubule dynamics and stabilizing microtubules in a manner similar to, but significantly weaker than that of clinically used microtubule targeting drugs [45]. Here, to understand and compare the antiproliferative and microtubule-targeting effects of erucin with those of sulforaphane, we analyzed the effects of erucin on proliferation, cell cycle progression, mitosis, apoptosis, and dynamic instability in MCF7 breast cancer cells stably transfected with enhanced green fluorescent protein EGFP-α-tubulin. We also analyzed the effects of erucin on the polymerization and dynamics of microtubules assembled from purified tubulin *in vitro*. We found that erucin inhibits proliferation of MCF7 cells in parallel with inhibition of cell cycle progression at G2/M and mitosis in a manner consistent with impairment of spindle microtubule dynamics. In addition, erucin strongly suppressed microtubule growing and shortening dynamics during interphase without affecting the microtubule polymer mass and did so in similar fashion with microtubules made from purified tubulin *in vitro*. Erucin also enhanced acetylation of microtubules in the cells, a marker for microtubule stabilization. These results strongly support the hypothesis that suppression of microtubule dynamics by erucin is important in its ability to prevent cancer.

## Materials and Methods

### Reagents

All chemicals were purchased from Sigma-Aldrich (St. Louis, MO) unless otherwise noted. Erucin (LKT Laboratories, Inc., St. Paul, MN) was dissolved in 100% dimethylsulfoxide (DMSO) and stored at −20°C.

### Cell Culture

Human MCF7 breast adenocarcinoma cells (American Type Culture Collection, ATCC # HTB 22, Manassas, VA), expressing EGFP-α-tubulin (Clontech, Palo Alto, CA [43] were cultured in 5% CO_2_ in standard glucose Dulbecco’s Modified Eagle’s Medium, with 10% fetal bovine serum (FBS, Atlanta Biologicals, Inc., Flowery Branch, GA ), 1% penicillin-streptomycin, non-essential amino acids, pH 7.3, 37°C. MCF7-EGFP-α-tubulin cells were morphologically indistinguishable from MCF7 cells. The population doubling time of the cells was 34 hours, as compared with 29 hours for unmodified cells. MCF7-EGFP-α-tubulin cells are referred to as MCF7 cells.

### Cell Proliferation

Cell viability was assessed by a modified sulforhodamine B assay (SRB ) [44]. Briefly, cells were seeded at 2×10^4^ cells per well in 96-well plates and incubated for 24 hours to allow cell attachment and recovery, incubated with erucin or vehicle control for 72 hours, fixed, stained, and the optical density (OD) of each well was measured (490 nm; Victor^3^V Wallac 1420 Spectrophotometer, Perkin-Elmer, Waltham, MA). The erucin concentration that inhibited proliferation by 50% (anti-proliferative IC_50_) was calculated as follows: 100– [100 (OD sample at 72 hours - OD control at zero time)/(OD control at 72 hours - OD control at zero time)]. Results are the mean and SEM of at least three independent experiments.

### Mitotic Index, Cell Cycle Analysis, Apoptosis

Cells were seeded at 3×10^4^ cells per well (volume, 2 ml) into six-well plates for 24 hours. Then the culture medium was replaced with erucin or vehicle control for 24 hours. Both floating and attached cells were collected and fixed in 10% formalin (25°C, 30 minutes), followed by cold methanol (4°C, 10 minutes). Fixed cells were mounted on glass slides with ProLong Gold antifade reagent containing DAPI [4,6-diamidinophenylindole] (Invitrogen, Eugene, OR), and examined by immunofluorescence microscopy (Nikon Eclipse E800, Melville, NY). The percentage of mitotic cells was determined by counting at least 500 cells for each condition.

Cell cycle analysis was performed using a Guava EasyCyte flow cytometer (Guava Technologies, Inc., Hayward CA) equipped with Cytosoft software 2.0. Briefly, non-synchronized MCF7 cells were seeded at 6×10^4^ cells in 2 ml for 24 hours, and incubated in the absence or presence of erucin for an additional 24 hours, harvested, fixed in cold 70% ethanol and stained using Guava Cell Cycle Reagent (Guava Technologies, Inc., Hayward CA) according to the manufacturer’s protocol. The DNA content of at least 5000 cells was measured for each condition. Cell cycle distribution was analyzed by ModFit LT 3.1 software (Verity Software House, Inc., Topsham, ME). Results are the mean and SEM of at least three independent experiments.

To determine populations of viable, dead, and apoptotic cells in the presence of erucin, cells were incubated with erucin for 24 or 48 hours. Apoptosis was determined using a Guava Nexin kit according to the manufacturer’s protocol on a Guava EasyCyte flow cytometer (Guava Technologies, Inc., Hayward CA). Data were analyzed using Guava Technologies software. Results are the means and SEM of at least three independent experiments for each condition.

### Immunofluorescence Microscopy

Cells were seeded on poly-L-lysine-treated coverslips at 6×10^4^ cells in 2 ml per well in six-well plates for 24 hours, incubated with erucin or vehicle control (24 hours), fixed in 10% formalin in PBS (20 minutes, 25°C) followed by 10 minutes in methanol (4°C), washed with PBS, and incubated with 1% normal goat serum (30 minutes). Cells were then incubated with mouse DM1 anti-α-tubulin antibody (1∶1000, Sigma-Aldrich, St. Louis, MO ) for 1 hour at 37°C, rinsed 3x in PBS containing 1% bovine serum albumin followed by incubation with goat anti-mouse FITC-conjugated secondary antibody (1∶1000, Sigma-Aldrich, St. Louis, MO) for 1 hour at 37°C. After washing, coverslips were mounted on glass slides with ProLong Gold antifade reagent with DAPI (Life Technologies, Grand Island, NY) to visualize nuclei, and cells were imaged with a Photometrics CoolSNAP HQ digital camera (Tucson, AZ).

### Analysis of Microtubule Acetylation

To analyze the degree of microtubule acetylation, cells were treated and fixed as above and the microtubules were visualized with rabbit anti-α-tubulin primary (1∶400, Sigma-Aldrich, St. Louis, MO) and secondary donkey anti-rabbit Rho (1∶150, Jackson ImmunoResearch Laboratories, Inc., West Grove, PA) antibodies. Acetylated microtubules were visualized with a mouse anti-α-acetylated tubulin primary antibody (1∶500, Sigma-Aldrich, St. Louis, MO) and a secondary goat anti-mouse-FITC-conjugated antibody (1∶1000, Sigma-Aldrich, St. Louis, MO). The extent of microtubule acetylation in the absence and presence of 15 µM and 30 µM erucin was analyzed with ImageJ software. Specifically, the fluorescence intensity of acetylated microtubules (green channel) in at least 25 interphase MCF7 cells per condition was analyzed according to the formula: corrected total cell fluorescence (CTCF) = Raw mean integrated density – (area of a selected cell × mean fluorescence intensity of background). Only flat and well spread cells were analyzed. Mitotic cells could not be analyzed because they were too small and round with the stain concentrated in small areas. High concentrations of erucin (50 µM and above) induced appreciable microtubule depolymerization and the majority of the cells were also round, thus preventing accurate measurement.

### Live Cell Imaging and Analysis of Microtubule Dynamic Instability in Interphase MCF7 Cells

Analysis of microtubule dynamic instability was carried out as described previously [45]. MCF7-EGFP-α-tubulin cells were seeded in six-well plates (6×10^4^ cells/2 mL/well) on poly-L-lysine, laminin, and fibronectin coated coverslips for 24 hours in culture medium with 10% FBS and then for 24 hours in culture medium with 2% FBS to induce flattening, after which erucin was added at the desired concentrations for an additional 24 hours. Video time-lapse images of microtubules in the thin peripheral regions of the cells were recorded as described previously [45]. Between 30 and 36 frames at 4-s intervals were taken with a 100X Nikon Plan Apo objective (37±1°C) on a Nikon Eclipse E800 microscope with a CoolSNAP HQ2 camera (Roper Scientific GmbH, Germany) under control of MetaMorph 4.9 software (Molecular Devices, LLC, Sunnyvale, CA). The positions of plus ends were tracked and graphed as microtubule length *vs*. time “life-history” plots, and the dynamic instability parameters were determined as described previously. Changes in length of ≥ 0.5 µm between two time points were considered growth or shortening events. Changes in length of <0.5 µm were considered periods of attenuated dynamics or pause. The catastrophe frequency is the frequency of transition from the growth or attenuated (paused) state to shortening. The rescue frequency is the frequency of transition from shortening to growth or the attenuated (paused) state. Dynamicity is the sum of all growing and shortening events divided by the total time measured, including time spent in the attenuated state.

### Purification of Microtubule Protein and Tubulin

Microtubule protein consisting of 70% tubulin and 30% microtubule associated proteins (MAPs) was isolated from bovine brains and MAP-depleted tubulin (purity >99%) was purified by phosphocellulose column chromatography and stored at −70°C as described previously [46]. For experiments, the purified tubulin was thawed and centrifuged at 4°C (35000 RPM) to remove any aggregated or denatured tubulin (Optima Max Ultracentrifuge, Beckman Coulter).

### Assembly of Microtubules and Determination of Steady-State Microtubule Polymer Mass

Purified MAP-free tubulin (2.75 mg/mL) was polymerized in PEM buffer [100 mM PIPES, 36 mM MES, 1.4 mM MgCl_2_, 1 mM EGTA, and 1 mM GTP pH 6.8] in the absence or presence of erucin for 35 min at 35°C, and then assembly was initiated with a suspension of sheared nucleating microtubule seeds prepared in 10% DMSO and 10% glycerol [45]. The final volume of seed suspension to tubulin solution was 1∶50. The reaction mixtures were polymerized for an additional 60 min at 35°C to achieve near steady state, and microtubule polymerization was monitored by turbidimetry at 350 nm using a Beckman DU 640 temperature controlled spectrophotometer. To determine the microtubule polymer mass, microtubules assembled as above reaction were pelleted by centrifugation at 35000 RPM for 1 hour at 35°C (Optima Max Ultracentrifuge). Microtubule pellets were solubilized in 0.2 M NaOH at 4°C to determine protein concentration [45].

### Effects of Erucin on Dynamic Instability of Purified Microtubules

The dynamic instability behavior at plus ends of individual purified microtubules was analyzed by video-enhanced differential interference contrast microscopy as described previously [47]. Briefly, purified bovine brain tubulin was mixed with sea urchin axoneme seeds and polymerized to steady state at the ends of the seeds (30 minutes, 35°C) in PMME buffer [87 mM PIPES, 36 mM 2-morpholinoethanesulfonic acid, 1.8 mM MgCl_2_, 1 mM EGTA, 1 mM GTP, pH 6.8] in the presence or absence of erucin. Microtubules were recorded for up to 60 minutes per slide. Microtubule lengths were measured every 3–5 s, plotted as length versus time, and analyzed using Real-Time Measurement software as described in [45], [47].

## Results

### Erucin Inhibits MCF7 Cell Proliferation in Association with Inhibition of Cell Cycle Progression at G2/M and Inhibition of Mitosis

Erucin (Fig. 1A, inset) inhibited MCF7 cell proliferation in a concentration-dependent manner with 50% inhibition (the IC_50_) occurring at a concentration of 28 µM (72 hours of incubation, Materials and Methods, Fig. 1A, -•-). Inhibition of proliferation occurred at the G2/M stage of the cell cycle as determined by flow cytometry. Specifically, 29% of a control cell population was in G2/M, consistent with previously published results with these cells (Fig. 1B) [45]. After 24 hours of incubation, 50% of the cells were in G2/M at 5 µM erucin, 60% at 15 µM erucin, and 69% at 25 µM (Fig. 1B, black bars). These effects are very similar to those of sulforaphane, which inhibited MCF7 cell proliferation at an IC_50_ of 26 µM and induced 64% accumulation of cells at G2/M at a concentration of 25 µM [45].

Inhibition of cell cycle progression at G2M by erucin was associated with mitotic arrest (Fig. 1 A, -▪-). Specifically, half-maximal accumulation of cells in mitosis at 24 hours occurred at 13 µM erucin and maximal mitotic accumulation (approximately 32–33%) occurred at 25 µM erucin. The inhibitory effects of erucin on mitosis in MCF7 cells were similar to those reported previously for sulforaphane, which induced half-maximal accumulation of cells in mitosis at a concentration of 13 µM [45].

### Induction of Apoptosis by Erucin

Similar to sulforaphane, erucin induced apoptosis in the cells in a time- and concentration-dependent manner (Fig. 1 C, see [45]). Specifically, in control cells there were 3.7% and 4.2% apoptotic cells at 24 and 48 hours, respectively. However, in the presence of 25 µM erucin, a concentration approximately twice the IC_50_ for inhibition of mitosis, there was a 4.8- and 8.6-fold increase in the number of apoptotic cells at 24 and 48 hours, respectively compared with controls. The extent of apoptotic cell death increased with higher erucin concentrations, reaching approximately 25% at 24 hours and 45% at 48 hours compared with 3.7% (24 hours) and 4.2% (48 hours) in controls (Fig. 1 C).

### Effects of Erucin on the Interphase Microtubule Network and Morphology of Mitotic Spindles

**Figure 2:**
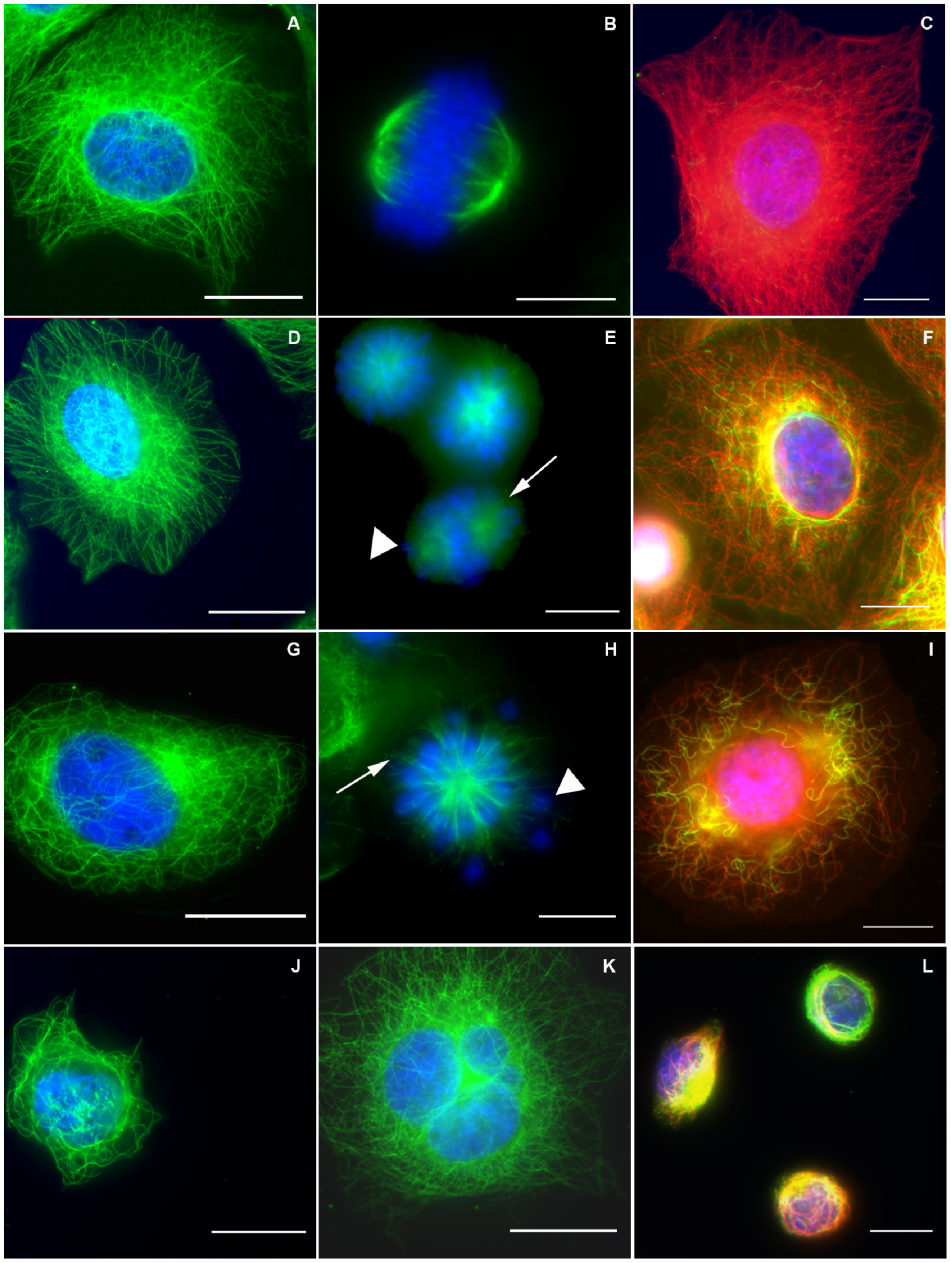
Concentration-dependence for the effects of erucin on cellular microtubule morphology, spindle morphology and microtubule acetylation in MCF7 cells. (**A**) Interphase control cells, (**D**) 15 µM, (**G**) 30 µM, (**J)** 50 µM, and (**K**) 25 µM erucin (a multinucleated cell). Control cells and cells incubated with 15 µM erucin show intact microtubule networks with similar overall morphologies. At 30 µM erucin, microtubules were somewhat shorter, reduced in number, and very curvy. At 50 µM erucin, the majority of microtubules were depolymerized with the remaining microtubules clustered around nucleus. Erucin also induced giant multinucleated cells (**K**, 25 µM erucin) at low and especially at high concentrations (data not shown). Metaphase spindles in control cells (**B**) were bipolar with all chromosomes congressed to the metaphase plate. The majority of cells arrested in mitosis by erucin displayed some abnormalities in metaphase: poorly defined bipolar spindles with uncongressed chromosomes ((**E**) 15 µM erucin, arrowhead) and an increased number of astral microtubules ((**E**) 15 µM erucin, arrows), or monopolar spindles that contained many uncongressed chromosomes ((**H**) 25 µM erucin, arrowhead). **A, B, D, E, G, H, J, and K**: chromosomes are blue, microtubules are green. **C, F, I, L:** acetylated microtubules in the presence of 0–100 µM erucin. Erucin promotes acetylation of microtubules in MCF7 cells in a concentration-dependent manner. (**C**) In control cells the majority of microtubules are dynamic (red color) with a very few acetylated microtubules (green microtubules). (**F**) At 15 µM erucin, there were more stabilized (acetylated) microtubules compared with those in controls. (**I**) At 30 µM erucin (approximately 2×mitotic IC_50_), there were fewer microtubules, and the majority of remaining microtubules were curved and acetylated (green). (**L**) In the presence of 50 µM erucin the majority of microtubules were depolymerized and strongly acetylated. Overall, the dynamics of microtubules was significantly stabilized by erucin, reducing the microtubule turnover. **C, F, I, L:** chromosomes are blue, microtubules are red, acetylated microtubules are green. Scale bar is 10 µm.

To determine the effects of erucin on the interphase microtubule network and on the morphology of mitotic spindles, MCF7 cells were fixed and stained for microtubules and chromatin and examined by immunofluorescence microscopy (Fig. 2, Materials and Methods). Microtubules in control interphase cells formed a fine filamentous network throughout the cytoplasm that emanated from the microtubule-organizing center (Fig. 2 A). Erucin concentrations as high as 15 µM (24 hour incubation) did not change the interphase microtubule organization detectably and the microtubules themselves were indistinguishable from those in control interphase cells (Fig. 2 D). In contrast, in the presence of 30 µM erucin, the concentration that produced approximately maximum mitotic accumulation, there were fewer microtubules than in control cells and those that remained were short and curvy (Fig. 2 G). At high erucin concentrations (≥50 µM) most cells became rounded and the microtubules were short and partially depolymerized (Fig. 2 J). These effects are very similar to those produced by sulforaphane [45] and resemble those of other microtubule-depolymerizing agents like the vinca alkaloids (see Discussion [42]).

Mitotic spindles in control cells displayed well-formed bipolar mitotic spindles with all chromosomes in metaphase cells congressed to a compact central region between the two well-separated spindle poles (Fig. 2 B). Normal spindles also displayed very few astral microtubules at the spindle poles. Erucin induced structural and functional spindle abnormalities at concentrations that did not detectably alter interphase microtubule organization. At 15 µM erucin, approximately 5% of the metaphase spindles appeared normal with properly duplicated and separated centrosomes, a compact metaphase plate with no lagging chromosomes, and very few astral microtubules at the spindle poles (data not shown). Approximately 25% of the spindles were still bipolar at 15 µM erucin, but they displayed abnormalities such as having several uncongressed chromosomes located at the spindle poles (Fig. 2 E, arrowhead). In contrast to mitotic spindles in control cells, which contained only a few astral microtubules, erucin-treated spindles displayed a relatively high number of astral microtubules (Fig. 2 E, arrow). Approximately 70% of the arrested mitotic cells displayed chromosomes that were arranged in a ball surrounding monopolar spindles in which centrosomes were not separated properly (Fig. 2 E). At 25 µM erucin, the concentration that induced the highest degree of mitotic arrest (mitotic index of 32.5%), monopolar spindles with uncongressed chromosomes predominated (Fig. 2 H, arrowhead), the spindle microtubules emanated from the center of monopolar spindles (Fig. 2 H, arrow), and the number of giant multinucleated cells increased to approximately 15.6% (13 fold) compared with 1.2% in controls (Fig. 2 K). The mitotic spindle aberrations induced by erucin were very similar to those induced by sulforaphane [45]. Interestingly, microtubule-targeting drugs such as the taxanes and the vinca alkaloids produce similar spindle abnormalities (see Discussion, reviewed in [38]).

The ratio of the number of cells in anaphase to the number of cells in metaphase during mitosis is a reflection of cell passage through the metaphase to anaphase transition. The anaphase/metaphase ratio in control cells was 0.55. In contrast, at 15 µM erucin the anaphase/metaphase ratio was only 0.09, a 6-fold decrease, indicating that erucin strongly slowed or prevented cells from transiting from metaphase to anaphase. This is also occurred with sulforaphane, and is similar to what occurs with more potent microtubule targeting drugs such as taxol and the vinca alkaloids [48].

### Effects of Erucin on Microtubule Dynamic Instability and Turnover in Interphase MCF7 Cells

**Figure 3:**
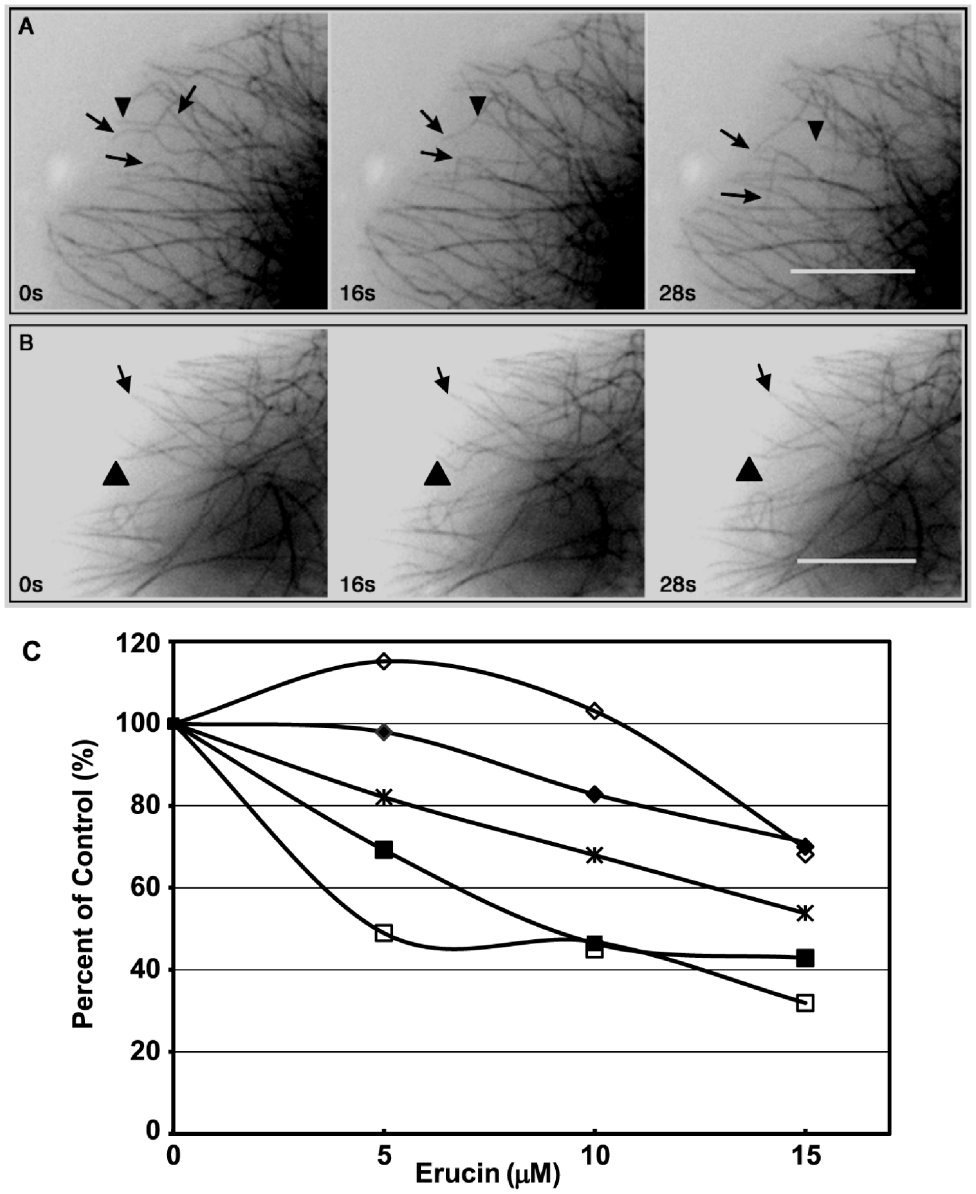
Dynamic instability of microtubules in MCF7 cells incubated with erucin. Time-lapse sequences of fluorescent microtubules at their plus ends: the positions of microtubule plus ends were tracked over time to generate life history plots in the absence (**A**) or presence (**B**) of 15 µM erucin, from which the dynamic instability parameters were calculated. Most microtubules in control cells grew (**A**, arrows) and shortened (**A**, arrowheads) within the course of the 2-min observation period. The dynamics were significantly suppressed by 15 µM erucin (**B**). Bar = 5 µm. (**C**) Concentration-dependent suppression of microtubule dynamics parameters in MCF7 cells by erucin: the growth rate (♦), growth length (◊), shortening rate (▪), shortening length (□), and dynamicity (×). Data are from Table 1.

While erucin at the IC_50_ for mitotic arrest did not detectably affect the organization and/or structure of microtubules during interphase, it did strongly affect the dynamic instability behavior of the microtubules. Typical images of interphase microtubules in control cells are shown in Fig. 3 A (top row panel) and microtubules in cells incubated with 15 µM erucin for 24 hours are shown in Fig. 3 B (bottom row panel). In control cells, the plus ends of microtubules (marked by arrows and an arrowhead) alternated in normal fashion among periods of relatively slow growth, rapid shortening, and pause, a state of attenuated dynamic activity that is below the resolution of the microscope. Microtubules in control cells grew at a mean rate of 14.5±1.07 µm/min, and shortened at 22.4±1.7 µm/min. Control cell microtubules also spent approximately 42% of their total time in the paused or attenuated state, neither growing nor shortening detectably (Table 1).

**Table 1:** Effects of Erucin on Dynamic Instability of Microtubules in MCF7-EGFP-α-Tubulin Breast Cancer Cells.

	ERUCIN (µM)						
PARAMETER	0	5	% Change	10	% Change	15	% Change
**Growth Rate (µm/min)**	14.5±1.0	14.2±1.0	**−**2	12.0±0.8	**−**17	10.3±0.6**	**−29**
**Shortening Rate (µm/min)**	22.4±1.7	15.5±0.7*	**−31**	10.4±0.8**	**−54**	9.6±0.7**	**−57**
**Growth Length (µm)**	3.3±0.3	3.8±0.3	**+**15	3.4±0.3	**−**3	2.3±0.1*	**−30**
**Shortening Length (µm)**	4.7±0.6	2.3±0.2**	**−51**	2.2±0.2**	**−53**	1.5±0.1**	**−68**
**Attenuation duration (min)**	0.27±0.02	0.35±0.04*	**+30**	0.35±0.02*	**+30**	0.38±0.04*	**+41**
**Time growing (%)**	29.5	39.7		40.0		35.9	
**Time shortening (%)**	28.6	28.3		22.5		20.1	
**Time attenuated (%)**	41.9	32.0		37.5		44.0	
**Catastrophe frequency, #/min**	2.0±0.2	1.8±0.2	**−**10	1.6±0.1*	**−20**	1.4±0.1**	**−30**
**Rescue frequency, #/min**	4.4±0.5	4.0±0.4	0	4.3±0.4	**−**2	5.6±0.4**	**+27**
**Dynamicity (µm/min)**	10.6±0.4	8.7±0.8*	**−18**	7.2±0.3**	**−32**	5.7±0.5**	**−46**
**Nondynamic microtubules (%)**	5	27		46		58	

Dynamicity per microtubule is the length grown and shortened divided by the total life span of the microtubule. At least 50 to 60 microtubules were analyzed for each condition. Values are means ± SE, except for the catastrophe and rescue frequencies which are means ± SD. Values were analyzed for statistical significance and differ significantly from controls at *P≤0.05, **P≤0.01 by Student’s *t*-test.

Erucin strongly suppressed the dynamic instability parameters of the microtubules in a concentration-dependent manner (Fig. 3 B, C). We quantified the effects of 5–15 µM erucin (24 hour incubation) on the dynamic instability behavior of interphase microtubules that displayed dynamic activity. Notably, approximately 60% of the microtubules were strongly stabilized at 15 µM erucin. As shown in Table 1 and Fig. 3 C, 15 µM erucin reduced the growth rate and length grown during a growth event by 29% and 30%, respectively. It suppressed the shortening rate and shortening length more effectively (by 57% and 68%, respectively). The time-based catastrophe frequency was decreased 30%, the rescue frequency was increased 27%, and the overall dynamicity was suppressed by 46% (Table 1). The effective suppression of dynamicity and stabilization of microtubules in MCF7 cells by erucin strongly resemble the effects of sulforaphane in these cells [45].

Acetylation of tubulin in microtubules is a marker for microtubule turnover and stability, with increased acetylation correlating with decreased turnover and increased stability [49]. The effects of erucin as visualized with an antibody against acetylated α-tubulin are shown in (Fig. 2 C, F, I, L). Few microtubules were acetylated in control cells, while incubation of cells with erucin for 24 hours resulted in a significant concentration-dependent increase in the extent of microtubule acetylation (Fig. 2 F, I, L, Fig. S1). Specifically, microtubule acetylation based upon the intensity of staining of acetylated tubulin in microtubules after treatment with 15 and 30 µM erucin increased approximately 3 and 4.4 fold, respectively, compared with that in control cells (Fig. S1). At high concentrations of erucin (50 µM), the majority of microtubules were depolymerized, and the remaining microtubules were highly acetylated and (Fig. 2 L), preventing the accurate assessment of acetylation in these cells. Similar results were obtained with sulforaphane [45] and with another microtubule-destabilizing drug, estramustine [50]. Thus, at erucin concentrations that inhibited mitosis but did not affect the quantity or arrangement of microtubules in the MCF7 cells during interphase (≤15 µM), erucin nevertheless suppressed the dynamics of the microtubules. It is reasonable to think that such suppression of microtubule dynamics perturbs the functions of interphase microtubules, such as their critical roles in cell migration and metastasis (see Discussion).

### Effects of Erucin on the Polymerization and Dynamics of Microtubules Assembled from Purified Tubulin

**Figure 4:**
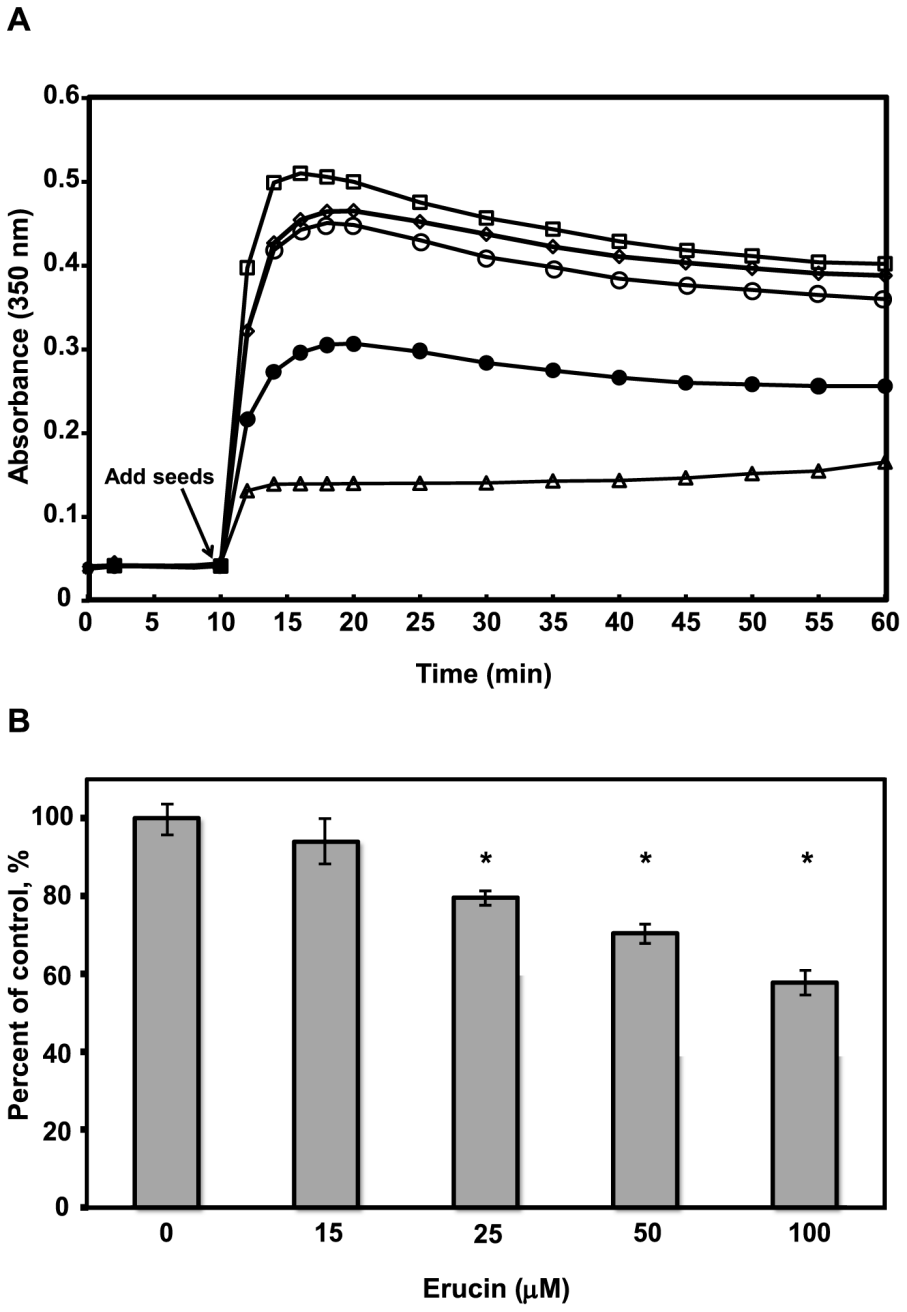
Erucin inhibits tubulin polymerization *in vitro*. (**A**) Polymerization of purified tubulin (2.75 mg/mL), pre-incubated with or without the drug for 35 minutes, was carried out at 35°C by initiation with nucleating microtubule seeds (Materials and Methods) in the absence (□) control, or presence of (◊) 15, (○**)** 25, (•) 50 µM, and (Δ) 100 µM erucin for 60 minutes. (**B**) Total microtubule polymer mass was determined by centrifugation 60 minutes after the initiation of assembly. Bars are ± SEM. Values with *are significantly different from control at ≥95% confidence interval by Student’s *t*-test.

Inhibition of microtubule polymerization by microtubule-destabilizing drugs (*e.g.* vinca alkaloids) requires much higher drug concentrations than those necessary to suppress microtubule dynamics [38], [42]. This also occurs with erucin. Erucin did inhibit polymerization of purified bovine brain tubulin into microtubules, but it did so relatively weakly (Fig. 4). Specifically, 15 µM erucin did not appreciably alter the rate or extent of microtubule polymerization (Fig. 4 A), while 25 µM erucin only partially inhibited the extent of polymerization (10.9±1%, not statistically significant, Fig. 4 B). Appreciable reduction of microtubule polymer mass required very high erucin concentrations (≥50 µM) with close to complete inhibition of polymerization occurring at 100 µM erucin (highest concentration tested, Fig. 4). Also, high erucin concentrations of ≥75 µM induced formation of tubulin aggregates as determined by electron microscopy (data not shown).

**Figure 5:**
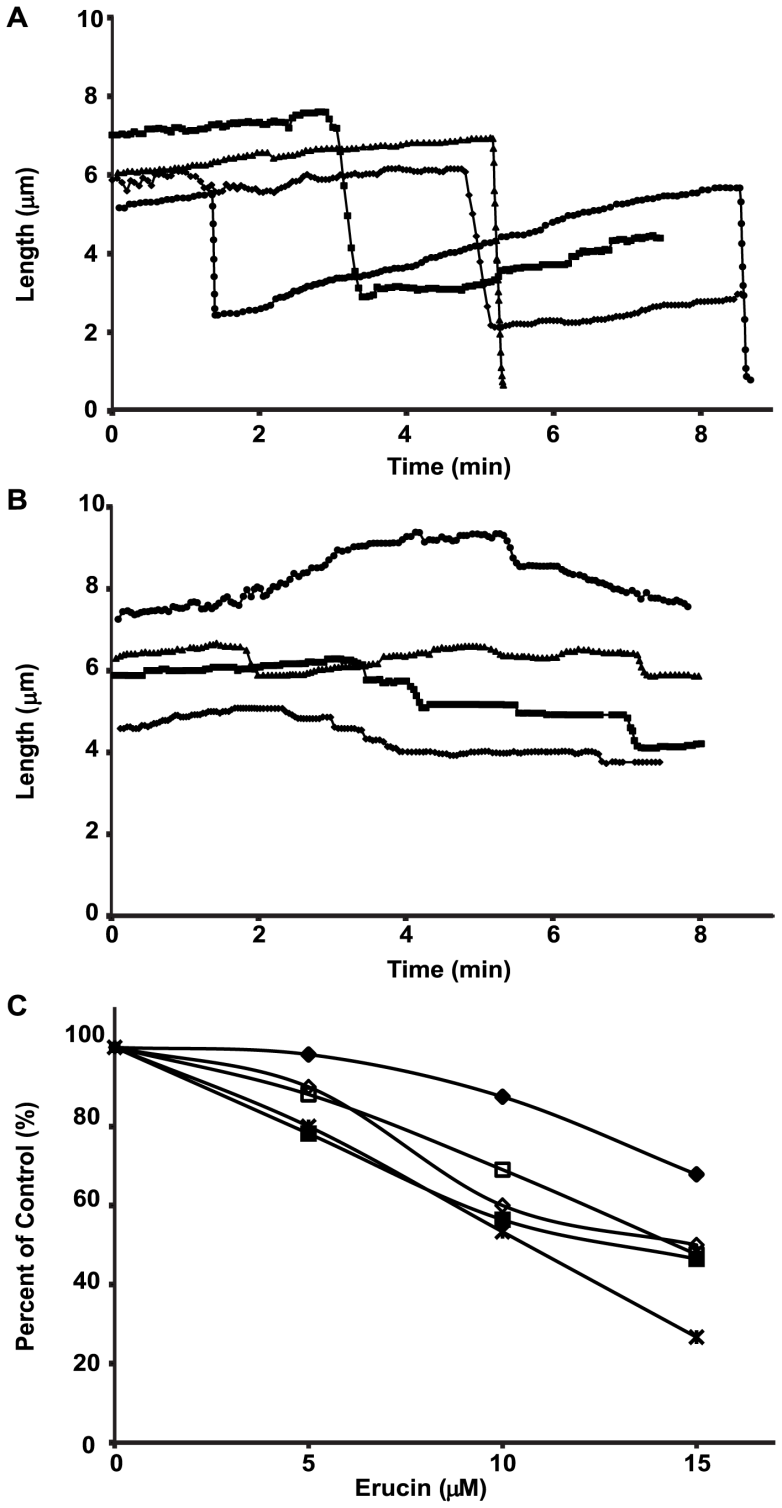
Concentration-dependent suppression of dynamic instability at microtubule plus-ends *in vitro* by erucin. Microtubule ends were tracked over time to produce life history plots of individual microtubules assembled *in vitro* from purified bovine brain tubulin. Life history plots are the length changes of individual microtubules in controls (**A**)**,** and in the presence of 15 µM erucin (**B**)**.** (**C**) Concentration-dependent suppression of microtubule dynamics parameters *in vitro* by erucin: the growth rate (♦), growth length (◊), shortening rate (▪), shortening length (□), and dynamicity (×). Data are from Table 2.

Similar to its actions in MCF7 cells, concentrations of erucin that were not high enough to affect the rate or extent of assembly of purified microtubules (≤15 µM erucin) strongly suppressed dynamic instability of these microtubules at their plus ends in a concentration-dependent manner (Table 2, Fig. 5, Materials and Methods). As shown in Fig. 5 A, control microtubules underwent slow growth and rapid shortening and spent relatively little time in an attenuated state (each line represents a single microtubule). In contrast, at 15 µM erucin, microtubules were strongly stabilized, growing and shortening more slowly than control microtubules, while spending more time in an attenuated (paused) state (Fig. 5 B).

**Table 2:** Effects of Erucin on Dynamic Instability of Steady-State Purified Microtubules *in Vitro*.

	ERUCIN (µM)						
PARAMETER	0	5	% Change	10	% Change	15	% Change
**Growth Rate (µm/min)**	0.56±0.1	0.55±0.2	**−**2	0.49±0.05	**−**13	0.38±0.02**	**−32**
**Shortening Rate (µm/min)**	21.1±4.6	16.5±2.9*	**−22**	11.9±3.5**	**−44**	9.80±1.7**	**−54**
**Growth Length (µm)**	1.0±0.1	0.9±0.1	**−**10	0.6±0.1**	**−40**	0.50±0.1**	**−50**
**Shortening Length (µm)**	4.2±0.5	3.7±0.2	**−**12	2.9±0.1**	**−31**	2.0±0.1**	**−52**
**Attenuation duration (min)**	0.9±0.1	0.9±0.2	0	1.2±0.1*	**+33**	1.5±0.1**	**+67**
**Time growing (%)**	68.7	59.8		38.2		32.1	
**Time shortening (%)**	8.5	11.0		12.3		12.9	
**Time attenuated (%)**	22.8	29.2		49.5		55.0	
**Catastrophe frequency, #/min**	0.3±0.1	0.3±0.2	0	0.26±0.1	**−**13	0.2±0.1*	**−33**
**Rescue frequency, #/min**	3.3±0.9	3.2±0.4	**−**3	3.70±0.5	**+**12	4.0±0.4*	**+21**
**Dynamicity (µm/min)**	1.5	1.2	**−20**	0.8	**−47**	0.4	−**73**

Tests of significance were not done on dynamicity, an overall variable. At least 25 to 35 microtubules were analyzed for each condition. Values are means ± SE, except for the catastrophe and rescue frequencies which are means ± SD. Values were analyzed for statistical significance and differ significantly from controls at *P≤0.05, **P≤0.01 by Student’s *t*-test.

Specifically, 15 µM erucin reduced the growth rate and the length grown during a growing event by approximately 32% and 50%, respectively (Table 2, Fig. 5 C). It also reduced the mean shortening rate by 54%, the length of a shortening excursion by 52%, the catastrophe frequency by 33%, and the rescue frequency by 21%. It increased the fraction of time the microtubules spent in an attenuated state (from 23% in controls to 55%) and it reduced the overall dynamicity of the microtubules by 73% (Table 2, Fig. 5 C). These effects of erucin on the dynamic instability parameters of purified microtubules are qualitatively similar to its effects on the dynamics parameters of microtubules in living interphase MCF7 cells, indicating that the effects of erucin on microtubule dynamics in cells are due to a direct action on the microtubules (Table 1, 2).

## Discussion

We have examined the antimitotic and antiproliferative mechanism of action of erucin, the major isothiocyanate present in Rocket salad species (*e.g*., arugula and wild rocket). We found that in MCF7 cells erucin acts very similarly to the structurally-related isothiocyanate sulforaphane [45]. Erucin’s main cellular effects are *(a)* inhibition of cell proliferation; *(b)* cell cycle arrest at G2/M; *(c)* suppression of microtubule dynamic instability in the interphase cytoplasm of the cells; *(d)* and induction of apoptosis. In addition, erucin’s effects on the dynamics and polymerization of microtubules made from purified tubulin were similar to those in cells, indicating that its effects on microtubule dynamics in cells are due to a direct action on the microtubules.

### Effects of Erucin on Mitotic Spindles

Erucin inhibited proliferation of MCF7 cells in a concentration-dependent manner with an IC_50_ of 28 µM, and it arrested cell cycle progression at mitosis with an approximately similar IC_50_ (13 µM), suggesting that inhibition of proliferation by erucin is associated with inhibition of mitosis. Similar to the action of sulforaphane, inhibition of mitosis by erucin appears to be caused by suppression of spindle microtubule dynamics. Inhibition of mitosis by erucin occurred at prometaphase/metaphase and was accompanied by formation of abnormal bipolar and monopolar spindles with improperly distributed chromosomes (Fig. 2). For example, at 15 µM erucin, many arrested mitotic spindles had a nearly normal complement of microtubules, but had one or several chromosomes remaining at the spindle poles unable to move to the metaphase plate (Fig. 2 E). Monopolar spindles predominated at 25 µM erucin, the concentration that induced maximum mitotic accumulation (mitotic index approximately 32%) (Fig. 2 F), indicating that at this concentration, cells were unable to build and/or maintain a functional bipolar spindle.

Significantly, cells arrested at mitosis by erucin, which displayed abnormal chromosome and spindle microtubule organizations similar to those induced by sulforaphane [45], were also similar to the abnormal spindles induced by more potent microtubule-targeting anticancer drugs such as taxanes and the vinca alkaloids [42], [51]. Suppression of spindle microtubule dynamics by these drugs causes several well-documented effects on mitotic spindle organization and function [38], [41]. They perturb spindle microtubule organization and prevent the chromosomes from properly attaching to the spindle microtubules and moving to the metaphase plate. For example, in Hela cells low concentrations of vinca alkaloids (0.2–30 nM) induce formation of spindles with longer, and more prominent astral microtubules than those of normal spindles, often with one or more chromosomes at one or both spindle poles rather than at the metaphase plate [41]. The same occurs with the taxanes [52], maytansinoids [53], and eribulin [39].

Suppression of dynamics by chemotherapeutic microtubule-targeted drugs reduces the tension at the kinetochores of the chromosomes and the cells fail to satisfy the mitotic spindle assembly checkpoint. Cells undergo a prolonged mitotic arrest and/or aberrant mitotic exit associated with formation of multinucleated cells and eventually die by apoptosis [51], [54]. Indeed, similarly to microtubule-targeted drugs and its analog sulforaphane, erucin induced the formation of multinucleated cells with the number of such cells increasing 13-fold at 25 µM erucin (15.6% compared with 1.2% in controls). Erucin treatment also resulted in a time- and concentration-dependent induction of apoptosis, with 25 µM erucin increasing apoptosis from approximately 4% in controls to 18% at 24 hours and to 31% at 48 hours.

### Effects of Erucin on Microtubule Polymerization and Dynamic Instability

In further support of the idea that erucin arrests cell proliferation at mitosis by perturbing microtubule dynamics, we determined directly that it suppressed microtubule dynamic instability in the peripheral regions of MCF7 cells during interphase. Specifically, 15 µM erucin (a concentration close to the IC_50_ for inhibition of mitosis), inhibited most dynamic instability parameters normally displayed by microtubules, including the rates and extents of growth and shortening and the transition frequencies between growth and shortening (Table 1). Erucin also increased the extent to which interphase microtubules were acetylated (Fig. 2 F, I, L; Fig. S1), an indicator of decreased microtubule turnover and stabilized dynamics [55], [56].

Erucin also suppressed the dynamic instability of microtubules made from purified tubulin *in vitro* in a manner almost indistinguishable from the way it suppressed microtubule dynamic instability in MCF7 cells. Both, the growth and shortening parameters and the dynamicity were suppressed *in vitro* by low concentrations of erucin (5–15 µM) (Table 2, Fig. 5). These results indicate that erucin is acting in cells directly on the microtubules themselves without substantially modifying the soluble tubulin levels, which would change the total polymer mass.

High concentrations of erucin (≥50 µM) did inhibit microtubule polymerization both in cells and *in vitro* (Fig. 2 J, L; Fig. 4 A, B), but the concentrations required to inhibit polymerization were much higher than those required to arrest mitosis and suppress dynamics of the microtubules in cells and to suppress the dynamics of purified microtubules (Table 1, 2; Fig. 5). This ability of erucin to suppress microtubule dynamics at concentrations well below those required to inhibit or increase the polymer mass, is mechanistically similar to the way the taxanes, the vinca alkaloids and many other microtubule targeting anti-mitotic agents, including sulforaphane, act. These agents suppress microtubule dynamics at concentrations below those required to increase (as with the taxanes) or decrease (*e.g*., eribulin, the vinca alkaloids) the microtubule polymer mass.

Among the anti-mitotic microtubule-targeted drugs that suppress microtubule dynamics (*e.g*., vinca alkaloids and taxanes), erucin’s specific actions on the various dynamics parameters, despite its much lower toxicity, most closely resemble those of vinblastine. Both erucin and vinblastine inhibit microtubule growth and shortening, while several other microtubule-depolymerizing drugs such as vinflunine and eribulin do not appreciably inhibit shortening [42].

While the mechanism by which erucin binds to tubulin or microtubules remains to be determined, one possibility is that it acts by covalent modification of a critical sulfhydryl residue of tubulin [57]. Indeed, a study by Mi *et al*. demonstrated that cysteines in tubulin are covalently modified by several isothiocyanates, including sulforaphane [58].

### Erucin as a Possible Anticancer and Chemopreventive Agent

Consumption of cruciferous vegetables, especially broccoli, broccoli sprouts and arugula is highly prevalent in the human diet. It seems clear that the anticancer and chemoprotective actions of erucin and sulforaphane occur at concentrations that are achievable in the human diet (from 0.1 to 300 µM, reviewed in [15], [59]). Isothiocyanates, including erucin and sulforaphane, are rapidly taken up into cells (to millimolar levels) as glutathione [GSH] conjugates [60]. Erucin and sulforaphane have similar absorption rates, elimination rates of their respective metabolites, and similar average bio-availabilities [15], [61].

It is interesting that that erucin and sulforaphane [45] exhibit microtubule-targeting activities that are qualitatively similar to those exerted by highly cytotoxic microtubule-targeting anticancer drugs, including taxanes, vincas, eribulin, and maytansinoids. However, when ingested at normal levels in cruciferous vegetables, isothiocyanates are not toxic. The reason for the lack of toxicity is probably due to the relatively low potency of the isothiocyanates as compared with the more powerful microtubule targeting drugs. Specifically, impairment of cellular microtubule functions in MCF7 cells occurs in the nanomolar range with taxanes and the vinca alkaloids, as opposed to the micromolar range with the isothiocyanates, a 1000 fold potency difference [33].

Recent evidence indicates that isothiocyanates can exert their anticancer activity alone and in combination with chemotherapeutic microtubule-targeting drugs to synergistically enhance cancer cell death [62], [63]. It seems reasonable to suggest that suppression of microtubule dynamics and impairment of highly sensitive microtubule functions by erucin and other isothiocyanates may be important in preventing and or retarding proliferation or migration of pre-malignant and/or malignant cells.

## Supporting Information

Figure S1 **Effects of erucin on microtubule acetylation in MCF7 cells.** Cells were incubated in the presence or absence of erucin for 24 hours (Materials and Methods). The fluorescence intensity of acetylated microtubules in at least 25 interphase MCF7 cells per condition was analyzed (A.U., arbitrary units). Results are from at least three independent experiments. Bars are ± SEM. Values with *** are significantly different from control at ≥99.9% confidence interval by Student’s *t*-test.
(TIF)
