# Supplementary figures and images for: Erucin, the Major Isothiocyanate in Arugula (Eruca sativa), Inhibits Proliferation of MCF7 Tumor Cells by Suppressing Microtubule Dynamics
Source: PLoS One. 2014 Jun 20;9(6):e100599. doi: 10.1371/journal.pone.0100599 (PMC4065051; doi:10.1371/journal.pone.0100599)

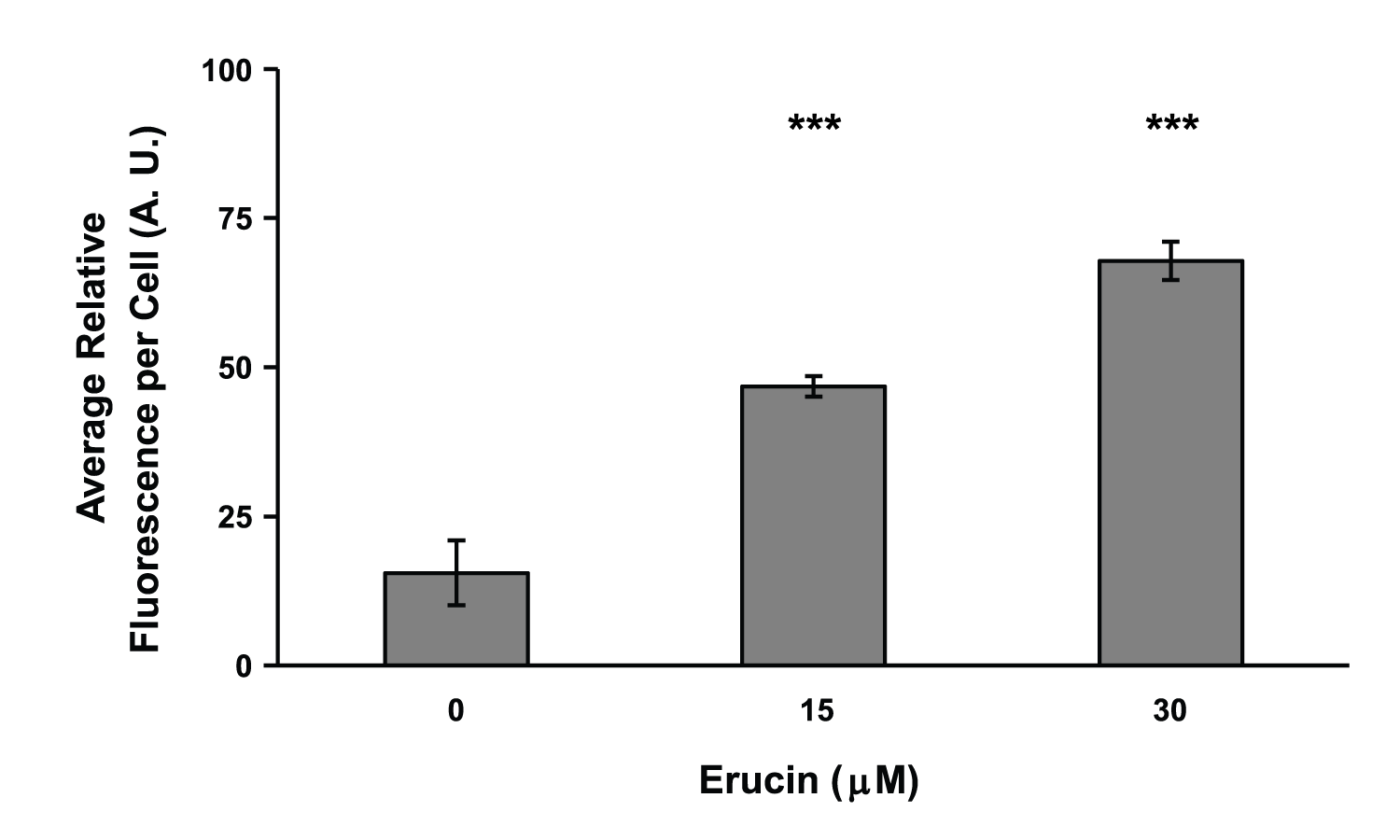

Supplement: Figure S1 — Effects of erucin on microtubule acetylation in MCF7 cells. Cells were incubated in the presence or absence of erucin for 24 hours (Materials and Methods). The fluorescence intensity of acetylated microtubules in at least 25 interphase MCF7 cells per condition was analyzed (A.U., arbitrary units). Results are from at least three independent experiments. Bars are ± SEM. Values with *** are significantly different from control at ≥99.9% confidence interval by Student’s t-test. (TIF) [file pone.0100599.s001.tif]
